# Animal Magnetism

**Published:** 1841-12

**Authors:** 


*Animal Magnetism, Dr. Collyer and his boy Frederick.*—We have heard much during the last four or five years about animal magnetism, and although we had heard statements concerning its wonderful effects, from gentlemen of the highest intelligence and veracity, we were nevertheless sceptical upon the subject, and having recently had an opportunity, during the visit of Dr. Collyer, and his boy Frederick, to Baltimore, of investigating the pretensions of mesmerism, our doubts have been resolved into a thorough conviction that it is nothing more nor less than a gross imposition.

Dr. Collyer failed in almost every thing which he attempted, except that of putting his subject into a mesmeric sleep, and that this was feigned, the boy gave the most indubitable indications, on the application of certain tests that were had recourse to by some of the gentlemen on the committee of investigation. For example, the holding of a small bottle of volatil ammonia to his nose, caused him to raise his head suddenly, this was repeated and with the same effect, and on applying capsicum to his mouth, certain jerkings and twitchings of his muscles and contortions of his face, gave manifest indications that, at least, the nerves of his mouth were not insensible to its effects. Seeing this, Dr. Collyer, said he was in a convulsion and soon proceeded to awake him from his magnetic sleep, by certain upward passes of his hand over his face. One evening the magnetized subject, answered several questions, by *mistake,* we suppose, that were propounded to him by two of the gentlemen of the committee, and on another occasion, while Dr. Collyer, was standing behind him, elevating and lowering his left arm, a gentleman present, Dr. Bond, requested Dr. Collyer to ask him how that tasted, the Dr. however, continued to work his arm, without complying with the request, but Frederick soon began to move his lips, and said, "dont like it, taste bad." Out of perhaps some thirty or forty questions that were put to Frederick, his replies except in two or three instances, were such, as we should suppose would convince any one, that animal magnetism was nothing but a vile and palpable imposition. We believe that Dr. Collyer, himself, is so fully convinced of this, that he will never renew his attempt to convince the people of Baltimore, that it is any thing else, though notwithstanding, he had the audacity to assert on the last evening of his exhibition, that the miracles wrought by our Saviour, were nothing more than perfect specimens of animal magnetism. We are gratified to perceive that the newspaper, as well as the medical press, with but very few exceptions, both in this country and in Europe, is lending its aid to put down this ridiculous humbug, and holding up to merited scorn and contempt, those who are using it for money-making purposes.—*Balt. Ed.*

